# Calcium Antagonists Use and Its Association with Lower Urinary Tract Symptoms: A Cross-Sectional Study

**DOI:** 10.1371/journal.pone.0066708

**Published:** 2013-06-21

**Authors:** Elsamaul S. Elhebir, Jeffery D. Hughes, Samantha C. Hilmi

**Affiliations:** 1 School of Pharmacy, Curtin Health Innovation Research Institute, Curtin University, Perth, Western Australia, Australia; 2 Pharmacy Department, Royal Perth Hospital, Perth, Western Australia, Australia; Northwestern University, United States of America

## Abstract

**Background:**

Lower urinary tract symptoms (LUTS) have been reported amongst the side effects of calcium antagonists (CA). CAs act on the bladder by affecting the ability of the detrusor muscle to create enough contractile force to overcome obstruction to normal voiding. We aimed to determine the relationship between CA use and LUTS in general medical inpatients.

**Methods and Findings:**

In this cross-sectional study we recruited 278 medical inpatients (including 85 CA users) aged ≥40 (72.1±13.7) years. LUTS was assessed using the International Prostate Symptoms Score (IPSS) questionnaire. A Logistic regression model using a ‘backwards-elimination’ strategy was used to identify variables associated with LUTS and for calculating the adjusted odds ratios and the 95% confidence intervals (CI). After adjusting for other risk factors and drugs, patients on amlodipine/nifedipine and diltiazem/verapamil (compared to non-users) were more likely to suffer from severe LUTS [Males: 12.45(CI: 1.57–98.63) and Females: 7.75(CI: 0.94–63.94)] and moderate-to-severe LUTS [Males: 17.43(CI: 2·26–134.39) and Females: 47.8(CI: 6.22–367.37)]. Patients on felodipine/lercanidipine were less likely to suffer from either severe or moderate-to-severe LUTS. Further, 19 (22.4%) CA-users were on treatment for LUTS compared to 18 (9.3%) of the non-users group, p = 0.003. Both male and female CA-users were three times more likely to be on alpha-blockers than non-users, p<0.001. CA-users were more likely to have undergone urinary tract-related surgery (Males: two times, p = 0.07 and females: nine times, p = 0.029). The study was limited by the fact that a causal relationship could not be established between CA use and LUTS.

**Conclusions:**

Our results demonstrate an association between CA use and an increasing severity of LUTS. They also demonstrate that CA-users are more likely to have medical or surgical treatment for LUTS. However, these CA’s effects on LUTS vary, and the use of highly vascular selective agents does not appear to pose significant risk.

## Introduction

Lower urinary tract symptoms (LUTS) consist of both storage and voiding related symptoms and include urinary hesitancy, poor stream, straining, frequency, incomplete bladder emptying, urgency, terminal dribbling, and nocturia. [1] A review by Chapple et al found that LUTS are very common and have similar prevalence in both men and women. [1] However, symptoms vary between males and females. Their review also revealed that LUTS include a progressive, age-related, non-sex-specific, non-organ-specific group of symptoms.

Apart from their natural progression with age, LUTS can also be caused by a number of other factors, e.g. spinal injuries, spinal spondylitis, Parkinson’s disease, pelvic surgery, and diabetes. In addition, males may develop LUTS because of prostatitis, benign prostate hyperplasia, and prostate cancer, while females may develop LUTS after childbirth or as a result of post-menopausal urogenital changes. [2] Some drugs are known to cause LUTS by affecting the bladder contractions and the micturition process. One such group of drugs is the calcium antagonists (CAs). CAs act on the bladder by affecting the ability of the detrusor muscle to create enough contractile force to overcome obstruction to normal voiding. The regulation of smooth muscle tone depends on the amount of intracellular free calcium (Ca^2+^). Human and animal studies have demonstrated the significant role of Ca^2+^ influx through L-type calcium channels in the contraction of the bladder. [3], [4] Some inhibitors of L-type calcium channels (i.e. CAs) are very potent inhibitors of bladder contraction in vitro in several species including humans, and actually are more potent in the bladder than in most vascular preparations. [5] Correspondingly, Cav1.2 knock-out mice (murine smooth muscle α_1c_ subunit calcium channel knockout [SMACKO] mice) have a strong bladder phenotype (i.e. they displayed difficulties in urinating as a result of lack of rhythmic contractions and a reduction of contractile responses to external stimuli). [6] Therefore, the blockage of calcium channels in the bladder may affect the ability of the detrusor muscle to create enough contractile force to overcome obstruction to normal voiding. However, despite the widespread use of CAs, there is only limited information on their association with bladder dysfunction in patients. Theoretically, CAs may also have an effect on bladder outlet and thus cause storage symptoms. In addition, earlier studies have reported some natriuretic and possibly diuretic effects of CAs [7]–[11], which may eventually lead to storage symptoms.

Further, some CAs have anti-cholinergic activity [12] and may cause anti-cholinergic-like side effects e.g. constipation and urinary retention. A number of studies have investigated the potential use of CAs for the treatment of over active bladder (OAB), but these have produced mixed results. [13]–[17] In addition, verapamil (and to a lesser extent diltiazem) in the therapeutic range is also an alpha blocker. Alpha blockers are the most frequently used drug class to treat male voiding symptoms and their adverse effects include urinary urgency and urinary incontinence. Manufacturers of CAs have included voiding and storage LUTS amongst the reported side effects in their drug’s product information, however without strong supporting literature.

A retrospective observational study revealed a possible association between CA use and worsening LUTS in 38 Australian males in the primary care setting. [18] The study demonstrated a statistically significant increase in the mean International Prostate Symptoms Score (IPSS) after commencing CAs (higher IPSS means worse symptoms) from a mean of 3.13 (95% CI: 2.09–4.17) to 9.82 (95% CI: 7.83–11.80), p<0.001. The results were still significant after adjusting for the natural progression of LUTS with ageing (mean IPSS: 5.85; 95% CI: 4.26–7.45), p<0.001. The study also demonstrated a significant deterioration in urological quality of life (QoL) after commencing CAs. The deterioration in QoL was significantly correlated with the change in IPSS (Pearson correlation 0.595; p<0.001). [18] The study recommended the evaluation of LUTS symptoms for all men before and after commencing CAs.

To our knowledge, no study has been conducted to evaluate the association between CAs and LUTS in the hospital setting. Our aim was to determine if there was an association between CA use and LUTS amongst a group of general medical inpatients aged 40 years or older and if so, the influence of gender on this association.

## Methods

This study was a cross-sectional study involving two groups of patients (CA-users and CA-non-users). The CA-non-users group acted as a control group. The data were collected using structured interviews and by reviewing the participants’ medical records.

All patients aged 40 years or older admitted to Royal Perth Hospital general medicine wards between August 1^st^ and October 31^st^, 2008 were invited to participate in the study and provided with a patient information sheet. Patients who agreed to participate in the study and provided a written informed consent were included in the study. Patients were excluded from the study if they did not give written informed consent, could not read or converse in English, were deemed too unwell by their doctors, had cognition impairment, or were catheterised. Patients also were removed from the study if consent was withdrawn. The study was approved by the ethics committees of both Curtin University and Royal Perth Hospital.

A demographic questionnaire was developed to collect the following information: patient gender, age, reason and date of admission, medical and surgical history (including all known risk factors for LUTS), medication history, and CA use and duration. The International Prostate Symptoms Score (IPSS) questionnaire was used to collect information about patients’ urological status. [19], [20] Despite being developed as a tool for males with benign prostate hypertrophy (BPH), the IPSS is widely used and acknowledged as a validated, non-disease specific and non-gender specific questionnaire. [20], [21] IPSS measures seven symptoms of LUTS with scores based on symptom frequency ranging from 0 (not at all) to 5 (almost always). The maximum score, corresponding to the worst possible LUTS, is 35. The American Urological Association (AUA) classifies LUTS severity into three main categories according to IPSS: mild (less than 7), moderate (8–19), and severe (20–35).

Simple descriptive statistics (frequencies and percentages for categorical variables, mean and standard deviations for variables measured on a continuous scale) were used to summarise the profile of study participants. Any differences in profile between CA users and non-users were assessed using the Chi-square statistic (for categorical variables), or t-tests (for continuous variables). In the case that a continuous variable appeared to be heavily skewed, a non-parametric test was used. IPSS was treated as a linear scale because the sample size was adequate for the assumption of normality to be valid. A Logistic regression model was used to identify any variables associated with development of severe (or moderate plus severe) LUTS. This model was developed using a ‘backwards-elimination’ strategy, whereby all the selected independent variables including age, gender, and past medical history listed in Table 1 were initially included in the model, and then the least significant variable was dropped until all variables remaining in the model were associated with development of LUTS (p<0.05). Patients from the mixed CA group (i.e. those taking more than one CA) were excluded from the regression analysis. Pairwise interaction terms, between independent variables which remained in the model, were also examined for statistical significance, during model development. The final results of the model were presented as odds ratios, their 95% confidence intervals and p-values. The odds ratios are referred to as ‘adjusted’ to stress that they were obtained from a multivariate logistic regression model, so that the results for each independent variable are ‘after adjustment’ for each other variable included in the model. For all statistical tests, a p-value<0.05 was taken to indicate a statistically significant association. Statistical analysis was performed using the SPSS version 15.0 software package and SAS version 9.2 (SAS Institute, Cary, NC, USA 2008).

**Table 1:** Demographic characteristics.

	CA-users (n = 85)	CA non-users (n = 193)	*p* values
Age	70.7 (14)	75.1 (12.7)	0.016
Days before the interview	4.9 (4)	5.4 (4.5)	0.522
**Gender**			0.017
Men	37 (43.5%)	114 (59.1%)	
Women	48 (56.5%)	79 (40.9%)	
**Past medical and surgical history**			
Alcoholism	7 (8.2%)	28 (14.5%)	0.146
Appendectomy	19 (22.4%)	26 (13.5%)	0.064
Arthritis	22 (25.9%)	36 (18.7%)	0.172
Asthma	22 (25.9%)	26 (13.4%)	0.012
Atrial fibrillation	19 (22.4%)	37 (19.2%)	0.542
Bladder/urethra surgery	7 (8.2%)	2 (1%)	0.002*
BPH/Prostate cancer	6 (7.1%)	21 (10.9%)	0.321
Cholecystectomy	13 (15.3%)	25 (13%)	0.601
Chronic obstructive pulmonary disease	19 (22.4%)	28 (14.5%)	0.108
Congestive heart failure	10 (11.6%)	28 (14.5%)	0.54
Dementia	2 (2.4%)	5 (2.6%)	1.00*
Diabetes	38 (44.7%)	63 (32.6%)	0.054
Epilepsy	4 (4.7%)	11 (5.7%)	1.00*
Gastroesophageal reflux disease	24 (28.2%)	28 (14.5%)	0.007
Hernia repair	21 (24.7%)	18 (9.3%)	0.001
Hypercholesterolemia	34 (40%)	59 (30.6%)	0.125
Hypertension	76 (89.4%)	93 (48.2%)	<0.001
Hypothyroidism	8 (9.4%)	15 (7.8%)	0.647
Hysterectomy	11 (12.9%)	19 (9.8%)	0.242
Impaired mobility	3 (3.5%)	12 (6.2%)	0.565*
Ischemic heart disease	42 (49.4%)	80 (41.5%)	0.218
Obesity	14 (16.5%)	76 (39.4%)	<0.001
Osteoporosis	9 (10.7%)	15 (7.8%)	0.441
Parkinson disease	1 (1.2%)	3 (1.6%)	1.00*
Peptic ulcer	15 (17.6%)	17 (8.8%)	0.033
Recurrent urinary tract infections	4 (4.7%)	14 (7.3%)	0.426
Spinal injury	11 (12.9%)	24 (12.4%)	0.907
Spinal spondylosis	2 (2.4%)	2 (1%)	0.588*
Spinal stenosis	2 (2.4%)	5 (2.6%)	1.00*
Stroke	19 (22.4%)	45 (23.3%)	0.86
TURP	7 (8.2%)	14 (7.3%)	0.775
Urological admissions	5 (5.9%)	14 (7.3%)	0.676

Data presented as either mean (SD) or number (%).

* Fisher’s exact test.

## Results

The primary investigator approached 311 patients of whom 32 patients declined to participate or asked to be interviewed at a later time; the remaining 279 patients agreed to participate (provided a signed written informed consent form) and were subsequently interviewed. One patient opted to withdraw after completing the interview. Therefore, a total of 278 (151 male [54.3%] and 127 female [45.7%]) patients completed the study. The mean age of the participants was 72.1±13.7 years (95% CI: 70.4–73.8 years) with the range from 42 to 96 years. About 63% of the patients were older than 60 years and 80% older than 50 years. There was no statistically significant difference between the age of males (70.7±14.5 years) and females (73.7±12.6 years), p = 0.68. Nevertheless, the age difference between CA-users (70.7±14) and non-users (75.1±12.7) was statistically significant, p = 0·016. Table 1 shows the general demographic information for the participants and occurrence of potential risk factors for LUTS.

A total of 85 patients (30.6%) enrolled into the study were using at least one CA. The distribution of drugs reflected the current Australian prescribing trends where amlodipine was the most commonly prescribed drug in Australia in 2008 (40%) according to the Pharmaceutical Benefit Scheme data. [22] Table 2 shows the distribution of CAs prescribed.

**Table 2:** CAs used by the participants.

Class	CA	Number of Patients (%)
DHP	Amlodipine	39 (45.9%)
DHP	Felodipine	13 (15.3%)
DHP	Lercanidipine	12 (14.1%)
Non-DHP	Diltiazem	10 (11.8%)
DHP	Nifedipine	8 (9.4%)
Non-DHP	Verapamil	1 (1.2%)
Mixed	Amlodipine/Diltiazem	1 (1.2%)
Mixed	Felodipine/Verapamil	1 (1.2%)

More than 90% (77 patients) of CA-users had been using a CA for more than a year, with about a third of those having taken CAs for more than 5 years. For the purpose of this analysis CAs were further classified into the following three categories: (a) Non-dihydropyridines (DHP) (verapamil/diltiazem) (b) Highly Vascular Selective DHP (lercanidipine/felodipine) (c) Other DHP (amlodipine/nifedipine).

The average IPSS for all participants was 11.1±7.6. Males had a statistically significantly higher mean IPSS 12.2±8.2 compared to females 9.7±6.6, p = 0.007. Patients on CAs experienced significantly worse LUTS compared to non-users. The mean IPSS was 15.2±8.1 for CA-users compared to 9.3±6.6 for non-users, p<0.001. The significant difference was apparent in both males and females. Females’ mean IPSS was 7.4±5.0 for non-users and 13.7±7.0 for CA-users, p<0.001. Whilst for males, the mean IPSS was also significantly higher among CA-users at 17.3±8.9 compared to 10.6±7.3 for non-users, p<0.001.

The difference between males and females was evident for all the symptoms. Males reported higher IPSSs for all the symptoms except urgency. However, statistically significant differences were only seen for intermittency, weak stream, and straining (Table 3).

**Table 3:** Mean score of IPSS question related to the symptom (0–5 scale) by gender.

Symptoms	MalesMean (SD)	FemalesMean (SD)	p-value
**Incomplete emptying**	1.60 (1.64)	1.35 (1.64)	0.21
**Frequency**	2.03 (1.61)	1.80 (1.63)	0.24
**Intermittency**	1.64 (1.62)	1.01 (1.43)	0.001
**Urgency**	1.74 (1.75)	1.80 (1.72)	0.77
**Weak Stream**	1.56 (1.79)	0.95 (1.30)	0.002
**Straining**	1.19 (1.63)	0.50 (1.13)	<0.001
**Nocturia**	2.44 (1.41)	2.33 (1.33)	0.51

A statistically significant difference was observed between genders with more males suffering from moderate and severe LUTS i.e. IPSS ≥7 compared to females (Table 4). Males, compared to females, were also more likely to suffer from severe LUTS i.e. IPSS ≥19; males 29 (19.2%) compared to females 11 (8.7%), p = 0.013, and moderate-to-severe combined; males 105 (69.5%) and females 69 (54.3%); p = 0.009.

**Table 4:** LUTS Severity by Gender and CA use.

		Severity of LUTS			
		Mild N = 104	Moderate N = 134	Severe N = 40	*p*-value*
**Gender**	**Males**	46 (30.5%)	76 (50.3%)	29 (19.2%)	**0.007**
	**Females**	58 (45.7%)	58 (45.7%)	11 (8.7%)	
**CA-Use**	**CA non-users**	89 (46.1%)	85 (44.0%)	19 (9.8%)	**<0.001**
	**CA-users**	15 (17.6%)	49 (57.6%)	21 (24.7%)	

* *p*-values are calculated from Chi-Square statistics and compares severity profiles between groups (gender and CA-use).

CA-users were more likely to suffer from moderate-to-severe LUTS compared to non-users (Table 4). CA-users were also more likely to suffer from severe LUTS, 21 (24.7%) compared to 19 (9.8%) for the non-users; p = 0.001. The severity of individual symptoms was higher in CA-users based on symptoms scores with exception of straining (Table 5). However, LUTS severity appeared to be independent of age in this study (Pearson’s correlation r = −0.005, p = 0.94) and length of stay in the hospital before the interview (r = −0.03, p = 0.6).

**Table 5:** Mean symptom scores for CA-users and non-users.

Symptoms	CA Users Mean (SD)	Non-userMean (SD)	*p*-value
**Incomplete emptying**	1.91 (1.86)	1.31 (1.51)	0.005
**Frequency**	2.58 (1.61)	1.63 (1.55)	<0.001
**Intermittency**	2.06 (1.72)	1.04 (1.39)	<0.001
**Urgency**	2.81 (1.70)	1.31 (1.55)	<0.001
**Weak Stream**	1.75 (1.83)	1.08 (1.46)	0.001
**Straining**	1.08 (1.66)	0.79 (1.36)	0.122
**Nocturia**	3.04 (1.31)	2.10 (1.31)	<0.001

Logistic regression was conducted and the adjusted odds ratios (AORs) were estimated for all significant risk factors. All variables in Table 1 were included in the initial model. Table 6 and Table 7 show the AORs for severe and moderate-to-severe LUTS respectively for all the patients and by gender. None of the felodipine/lercanidipine users suffered from severe LUTS. Therefore, they are excluded from the analysis under the severe LUTS category. Amlodipine/nifedipine and diltiazem/verapamil use had shown a great association with both severe and moderate-to-severe LUTS in all patients.

**Table 6:** Adjusted odds ratios by logistic regression- Severe LUTS.

Dependent Variables	Independent Variable	Outcome (n/N)*	Odds ratio (95% CI)	p value
**All patients**	Gender (female)	11/127	0.24 (0.1–0.6)	0.001
	Alcohol	8/35	3.3 (1.2–9.4)	0.27
	Gender specific operation (hysterectomy and TURP)	15/54	5.1 (2.1–12.4)	<0.001
	Obesity	17/90	3.5 (1.5–8.1)	0.004
	Amlodipine/nifedipine	17/47	9.8 (4.0–24.3)	<0.001
	Diltiazem/verapamil	3/11	8.2 (1.9–34.9)	0.004
	Non-users	−/−	1 (Reference)	–
**Males only**	Alcohol	8/25	6.7 (1.9–23.4)	0.003
	Benign Prostate Hypertrophy	10/27	5.3 (1.7–17.2)	0.005
	Obesity	12/49	2.8 (1.0–7.6)	0.048
	Amlodipine/nifedipine	11/23	7.3 (2.4–22.3)	<0.001
	Diltiazem/verapamil	2/5	12.45 (1.6–98.6)	0.017
**Females only**	Alcohol	0/10	–	–
	Hysterectomy	7/33	5.9 (1.4–24.8)	0.039
	Obesity	5/41	1.55 (0.22–10.8)	0.758
	Amlodipine/nifedipine	6/24	10.5 (2.2–49.2)	0.003
	Diltiazem/verapamil	1/6	7.8 (0.9–63.9)	0.057

* (n/N) the number of patients with severe LUTS/Total number of patients exposed.

**Table 7:** Adjusted odds ratios by logistic regression-Moderate-to-severe LUTS.

Dependent Variables	Independent Variable	Outcome (n/N)*	Odds ratio (95% CI)	p value
**All patients**	Gender (female)	69/127	0.3 (0.2–0.6)	<0.001
	Gender specific operation (hysterectomy and TURP)	40/54	2.1 (1.0–4.6)	0.053
	Obesity	62/90	2.3 (1.3–4.3)	0.007
	Felodipine/lercanidipine	12/25	1.4 (0.6–3.4)	0.499
	Other CAs	56/58	37.5 (8.6–163.9)	<0.001
	Non-users	−/−	1 (Reference)	–
**Males only**	Obesity	39/49	2.5 (1.1–5.7)	0.035
	Felodipine/lercanidipine	5/9	1.0 (0.2–3.8)	0.945
	Other CAs	27/28	17.4 (2.3–134.4)	0.006
**Females only**	Obesity	23/41	1.43 (0.43–4.8)	0.564
	Felodipine/lercanidipine	7/16	1.2 (0.4–3.6)	0.725
	Other CAs	29/30	47.8 (6.2–367.4)	<0.001

* (n/N) the number of patients with moderate-to-severe LUTS/Total number of patients exposed.

As all the patients in the diltiazem/verapamil group had moderate-to-severe LUTS, the amlodipine/nifedipine and diltiazem/verapamil groups were combined together to form a new group called ‘other-CA’. After adjusting for gender, gender specific operations, and high body mass index, the AOR of having moderate-to-severe LUTS when using other-CA was significantly higher than the non-users. Patients on felodipine/lercanidipine have an AOR of 1.4 (95% CI, 0.6–3.4) indicating a potential lack of effect on LUTS of these agents. Table 6 and Table 7 show the gender differences for severe and moderate-to-severe LUTS.

An estimated 22.4% (19) of the CA-users group were taking treatments for a range of urinary symptoms compared to 9.3% (18) of the non-users group (Pearson Chi Square, p = 0.003). About 27% (10 of 37 patients) of male CA-users were on alpha-blockers compared to only 7.9% (9 of 114 patients) of non-CA-users, Fisher’s Exact Test p = 0.004. Likewise, five out of six of female CA-users were taking prazosin (10.4% of total females), which is uncommon as it is widely used for urinary retention (a problem usually linked to prostate enlargement), Fisher’s Exact Test p = 0.29. None of the patients in Non-DHP or Other DHP groups were taking treatment for OAB, e.g. oxybutynin and solifenacin. Nevertheless, four (16%) patients on Highly Vascular Selective DHP were on treatment for OAB compared to seven (3.6%) of non-users, Fisher’s Exact Test p = 0.021.

CA-users were also more likely to have undergone urogenital surgery 16.5% (14) compared to non-users 7.8% (15), Chi Square p = 0.029. Female patients who were receiving a CA were about nine times more likely to have undergone urogenital surgery 10.5% (5) than non-CA-users 1.2% (9), p = 0·029. While, male CA-users were two times more likely to have had urogenital surgery 24.3% (9) compared to non-users 12.3% (14), p = 0.07.

## Discussion

This study provides further evidence of an association between CA use and LUTS, and that this association exists for both males and females. The prevalence of severe and moderate-to-severe LUTS in this study appears to be higher than has been reported in previous international general demographic studies. [23]–[25] A South Australian study estimated the prevalence of bothersome LUTS to be at 26% for men and 39% for women (all ages) and 48% for both men and women over 65. [26] Another Australian study conducted by Muscatello et al in the Sydney metropolitan area showed a higher prevalence of 53% for men and 61% for women (all ages). [27] This study reported that for those over 60 years the prevalence is more than 75% for men and 66% for women. [27] The higher prevalence found in the current study may be due to the high mean age and the physical condition of the patients studied. However, contrary to previous studies, the data did not show an association between the severity of LUTS and age. This was an unexpected finding, and we can only speculate this might be due high proportion of participants being over 60 years old (about 63%). Previous studies reported higher prevalence in this age group. [27] The higher prevalence of LUTS in males is consistent with what has been previously reported in earlier studies. [1], [28]–[31] Males reported significantly higher obstructive symptoms e.g. intermittency, weak stream, and straining. A positive association was observed between CA use and the severity of LUTS. The average IPSS was significantly higher (about two-fold) for CA-users regardless of their gender. This difference was statistically significant for all symptoms other than straining. Based on the available data, the use of CAs in females appeared to be associated with a greater risk of more severe LUTS compared to their use in males. Our findings are supported by two recent studies which looked at the effects on antihypertensive medications on LUTS. The first study of 3790 Japanese men suffering from LUTS looked at the association between LUTS and hypertension. [32] The study revealed that patients on CAs had a statistically significantly higher IPSS 19.6 compared to 18.2 for patients with no treatment for hypertension, p<0.05. Patients on angiotensin receptor blockers scored significantly lower IPSS, while patients on angiotensin converting enzyme (ACE) inhibitors were shown to have scores not significantly different to the no-treatment group. [32] The other epidemiological study from Boston investigated the association between common antihypertensive medication use and LUTS in 5503 males and females. [33] The Boston area community health (BACH) survey demonstrated a high prevalence of LUTS in women CA users especially for those <55 years old (nocturia: OR = 2.65, 95% CI: 1.04–6.74; voiding: OR = 3.84, 95% CI: 1.24–11.87). Concurrent use of loop diuretics and CA was also associated with higher prevalence of LUTS in both males and females. However, CA use alone in men did not appear to increase LUTS. Hall et al also reported that the use of CAs in women, either alone or in combination with other antihypertensive agents was associated with an increased prevalence of LUTS (voiding issues and nocturia, but not storage symptoms), although these adverse effects appeared to be confined to those women aged <55 years. [33] In the other study by Ito et al, investigators found CA users had a significantly higher mean score compared to non-treated hypertensive patients. [32] The authors commented that contrary to what they had expected, “sub-scores for intermittency, not for storage symptoms, were worse in patients with CA treatment in this study.” This finding is consistent with that of Hall et al. Interestingly, in Ito et al’s study men taking angiotension receptor antagonists were found to have significantly lower IPSSs, yet there were no significant differences in quality of life across the antihypertensive classes.

In comparing the different CAs, patients on Highly Vascular Selective CAs did not appear to be at increased risk of LUTS compared to non-users, with no patients within this subgroup suffering from severe LUTS. The high vascular selectivity of felodipine and lercanidipine appears to be associated with lower bladder selectivity. Lercanidipine has been reported to have 177 times higher selectivity for the aorta compared to its bladder selectivity. [34] Unfortunately, an extensive literature search and correspondence with the manufacturer failed to locate any evidence on the bladder selectivity of felodipine. The paucity of literature evidence associating LUTS side effects to CAs use may lead to the neglect of such risk. Whilst, failure to consider CA use as a potential contributing factor may result in patients being investigated and/or treated for LUTS, which may have responded to drug withdrawal. This is supported by Williams and Donaldson, who based on their experience of withdrawing nifedipine in nine males referred for LUTS surgery (6 had complete symptom resolution and 3 considerable improvement) suggested that drug withdrawal should be considered before prostatectomy. [35] Amongst this group of patients, CA-users were more likely to be receiving medication to manage LUTS and/or to have undergone urogenital procedures.

Logistic regression confirmed the association between non-DHP (diltiazem, verapamil) and other DHP CAs (amlodipine, nifedipine) use and the existence of severe and moderate-to-severe LUTS. The odds ratio was statistically significant for both males and females. In addition, logistic regression also confirmed that Highly Vascular Selective CA-users (felodipine/lercanidipine) were no more likely to suffer from severe and moderate-to-severe LUTS compared to the non-CA users. The data also showed an increase use of OAB medications (e.g. oxybutynin) amongst patients on felodipine/lercanidipine while none of the other CA-users were taking OAB medication. It is not clear whether other CAs were in fact preventing/treating OAB as suggested by earlier literature or felodipine/lercanidipine were contributing to it. Further research is required to assess this association.

Given the widespread use of CAs, educating healthcare providers about the potential association between CAs and LUTS may help to prevent unnecessary interventions and potentially improve the QoL of their patients.

There are a number of limitations to our study. Despite showing a significant association between CA use and LUTS that was independent of all the risk factors considered, this cross sectional study could not confirm a causal relationship. Further, the relatively small sample size does raise the possibility of type II errors (i.e. the error of finding no association when one is present). However, our results are consistent with those of recent epidemiological studies suggesting an association between CA use and LUTS. [32], [33].

In conclusion, the study results have demonstrated a high prevalence of LUTS amongst both males and females, with males more likely to suffer from severe symptoms. CAs-users appear to suffer more severe LUTS than non-users. They are also more likely to undergo urinary related surgery and receive medical treatment for LUTS. However, the results have suggested that this association may not be a class effect. Therefore, we recommend all CA-users should be monitored for LUTS, and healthcare practitioners should consider CAs as a potential cause of LUTS and consider stopping/changing these drugs where appropriate. Finally, larger prospective randomised controlled trials are needed to further evaluate the association between different CAs and LUTS.
